# Post-growth economics: a must for planetary health justice

**DOI:** 10.1186/s12992-023-00957-2

**Published:** 2023-08-08

**Authors:** Winne Fleur van Woerden, Remco van de Pas, Joel Curtain

**Affiliations:** 1 Programcoordinator Degrowth & Care Economy, Commons Network, Amsterdam, Netherlands; 2 Universitat Autònoma Barcelona, Barcelona, Spain; 3 Centre for Planetary Health Policy, Berlin, Germany; 4 Institute of Tropical Medicine, Antwerp, Belgium; 5 Maastricht University, Maastricht, Netherlands; 6 Partners In Health, Boston, MA USA

**Keywords:** Planetary Health, Decolonization, Post-growth Economics, Ecological justice

## Abstract

Within the global health field, progress is being made to adopt a justice and sustainability-centred approach by advancing what has been named a planetary health agenda. Meanwhile, an increasing number of global health scholars argue for the decolonisation of the field. Yet, amongst these collective efforts to ‘transform’ global health thinking, a thorough analysis of political economy dimensions is often missing. ‘Growthism’, the belief that more production is necessarily good, continues to prevail. Truly committing to a decolonial eco-just global health agenda requires addressing the continuation of colonial arrangements within the structure of the global economy, removing growth dependencies and ushering in post-growth policies.

## Background

Vast and unequal capitalist accumulation of wealth over the past decades has triggered a process of global ecological deterioration that fundamentally undermines the foundation of human life as we know it [1–3]. A 2023 update of the planetary boundaries framework coined by Rockstrom and colleagues as “the safe operating space for humanity” showed that seven out of nine planetary boundaries are currently being transgressed, jeopardising the capacity of our planet to safeguard health and wellbeing [4]. Scientific research shows clearly that environmental changes are already affecting human health. These health impacts result from direct effects, through heat stress or flooding for example, and indirect effects, such as diminishing food yields, the spread of infectious diseases, and increased forced displacement and conflict [3, 5]. According to the World Health Organization (WHO), climate change is now “‘the single biggest health threat facing humanity’ in the 21st century [6].

The concept of ‘planetary health’ was coined in 2015 as a call to global health actors to broaden their view to pay attention to the “systems that shape the future of humanity and the Earth’s natural systems that define the safe environmental limits within which humanity can flourish” [1]. Planetary health is a broad and interdisciplinary field that expands focus from local environmental threats studied in the past towards the health consequences of changes on a planetary scale. Planetary health research commonly acknowledges that these health risks are not equally distributed and that communities in the global South are hardest hit by ecological collapse [1, 5, 7]. In their book *Planetary Health: Protecting Nature to Protect Ourselves*, Myers and Frumkin write: “fundamentally, planetary health places us in new ethical terrain. It teaches us that all people on this planet, those alive today and in the future, are connected to one another” [7].

Although the planetary health frame represents a significant advancement for ushering in a more holistic, sustainability-centred understanding of global health, we argue that its dominant discourse remains insufficient from a historical and political economy of health perspective. This becomes all the more problematic when such an ahistorical and apolitical analysis drives global health policy [8]. Global health actors need to incorporate a more comprehensive analysis of the political economy of health and ecology; in doing so, they will come to understand post-growth principles as indispensable in advancing human and planetary health. Adopting a post-growth frame means understanding the necessity to remove the constant pressure that Northern governments and companies apply to depress the costs of labour and resources in the South to fuel shareholder profits and increase wealth accumulation and consumption growth in rich nations. It means focusing our attention as global health advocates on dismantling the colonial relationships upheld by the growth imperative within our global economic policies, institutions and agreements [9].

## Post-growth policies and decolonial thought

Among those bringing eco-centred thought to the global health field some are already challenging the hegemony of economic growth, including in the medical domain, as the core driver of the ecological crisis [10–13]. Hensher et al., for example, write that nowadays a considerable part of healthcare in the global North can be described as ‘uneconomic’, whereby overconsumption of medical products and iatrogenic medical care induce environmental harms and social costs that are greater than the benefits of that investment [11]. Moon has pointed out that the powers that drive uneconomic growth in Western healthcare are also prominent in pushing biomedical innovations and ‘technical solutionism’ in the global South, including in preparing and responding to future pandemic risks [14]. Their analysis goes back to that of Ivan Illich, who, in the 1970s, highlighted the growing tendency to expropriate health in industrialised societies. If medicine is produced as if it were a commodity, then a larger by-product will become evident, namely the fallacy that, in the words of Illich, ’society has a supply of health locked away which can be mined and marketed’ [15]. In 2022, the 6th edition of the Global Health Watch dedicated a full chapter to degrowth, noting its potential to “create new social imaginaries to confront the still hegemonic, growth-based neoliberal capitalism” [16].

The field of global health, with its roots in colonial and tropical medicine, must contend with implicit and explicit supremacy attitudes, and hence as a discipline holds a deep internal contradiction as it aims to reduce inequities globally. As such, a fundamental reorientation of the discipline is required, including the decentralisation and democratisation of knowledge platforms and a deeper focus on international solidarity, local needs and sufficiency as drivers for tacit understanding and (policy) action [17]. This reorientation must be reflected within planetary health research and policy efforts as well if the field is to avoid reinforcing colonial and capitalist logic. Decolonial thought is indispensable in moving planetary health beyond the divide between Indigenous and western ontologies and countering “the tendency of mainstream environmentalism to erase the ongoing effects of colonialism and Indigenous Knowledges” [18, 19]. Moving toward the dual goals of equity and sustainability requires decolonisation, i.e. “the restoration of Indigenous land and life” as defined by Tuck and Yang [20]. Those within the global health community advancing the planetary health agenda should have at the forefront of their mind the words of the People’s Agreement of Cochabamba: “The corporations and governments of the so-called “developed” countries, in complicity with a segment of the scientific community, have led us to discuss climate change as a problem limited to the rise in temperature without questioning the cause, which is the capitalist system. . [which] has imposed on us a logic of competition, progress and limitless growth” [21].

Understanding the interconnectedness between the determinants of planetary health thus requires decolonial analyses. This move towards decolonisation, in turn, requires incorporating post-growth principles, especially in the realm of material and economic decolonisation. It would move the field towards centring the interconnectedness between the environment, the economy, and health while integrating the imperative of ecological economics (i.e. equitable meeting of human needs within planetary boundaries) with material decolonisation (i.e. exposing and tackling drivers of excess energy and resource use in rich countries and dismantling the current colonial arrangements in our global economic system).

## Post-growth: The required frame for decolonial planetary health thinking

The unprecedented ecological impact of human economic activity led to the labelling of the current epoch as ‘the Anthropocene’, which positions human action as the planetary-scale geological force that is determining today’s state of the Earth [22, 23]. The term is commonly quoted by planetary health scholars when bringing attention to global environmental changes [1, 24, 25]. However, the overshoot of planetary boundaries is not caused by humans as such - as the term ‘Anthro’ suggests. Rather, it is being driven by a particular global economic system that is built around and dependent on continued economic growth and injustice to the benefit of the wealthiest few living predominantly, but not only, in global North countries. To uphold this growth imperative and maintain Northern levels of production and consumption, the global economy relies on an imperial arrangement of cheapening and appropriating resources and labour from countries in the global South [26].

Research has indicated that the net appropriation of resources and labour from Southern to Northern countries through what is known as ‘unequal exchange’ represented a drain from South to North over the period 1990–2015 of $242 trillion (constant 2010 USD). Resources that could otherwise be mobilised to meet domestic needs in Southern countries are instead appropriated to service production and consumption in the global North, through processes such as deeply unequal trade relations, the migration of minds as well as human and financial capital, the exploitative business practices of transnational corporations, and technological dependencies that are unfairly protected via international Intellectual Property regimes. The same study found that the South’s losses due to unequal exchange outstripped their total aid receipts, including development assistance for health, by a factor of 30 [27].

These production and consumption patterns in the global North are responsible for the vast share of excess energy and resource use that is causing global ecological breakdown. The global North is responsible for 92% of global CO2 emissions in excess of the safe planetary boundary that are driving the climate crisis [28]. It is also responsible for 74% of global excess resource use, the predominant driver behind the overshoot of other boundaries and the Sixth Mass Extinction [29, 30]. Meanwhile, as widely acknowledged by planetary health scholarship, the health and economic burden of the ecological crisis is falling disproportionately on communities in the global South, particularly those who bear no responsibility for ecological breakdown [3].

By focusing on scaling down Northern material and energy use and reducing Northern consumption and production levels while redistributing wealth and income more fairly, post-growth principles and policies not only acknowledge the reality of differentiated responsibilities for the overshoot of planetary boundaries - they are also in line with breaking the colonial patterns of appropriation that underpin rich countries’ energy and resource use that is driving the ecological crisis [31]. As such, post-growth economics are not only in line with the planetary health agenda - it is also deeply rooted in decolonial thought that goes back to thinkers like Salvador Allende, Frantz Fanon, and Thomas Sankara, who all saw an autonomy-centred approach to development as paramount to throwing off neo-colonial power [32–34]. It builds upon the work of dependency theorists who have exposed how growth in the North depends on the appropriation of Southern resources and labour and have pointed out that capitalism has been responsible for the accentuation of underdevelopment hence the impossibility of “catch-up” development [35]. And it is rooted in core demands spelt out by United Nations member states diplomatic initiatives like the Non-Alignment Movement for a New International Economic Order (NIEO) to create space and autonomy for post-colonial Southern economies to shift away from their enforced role as exporters of cheap labour and raw materials and to focus instead on building economies centred around sovereignty, self-sufficiency, and human well-being [31, 36].

## A new political-economic framework for global health policy

The global health policy field today remains enmeshed in the ‘Investing in Health’ discourse enunciated in the 1993 World Bank report with the same name [37]. At its core, this approach promotes economic growth, International Financial Investments, technological innovation and Public-Private Partnerships as means to reduce poverty and deliver development goals, such as Universal Health Coverage. Through this discourse, ‘Sustainable’, ‘Inclusive’ and ‘Green’ Growth pathways are seen as crucial objectives to prevent and recover from global crises, including the COVID-19 pandemic and the climate crisis. Just like this framing is the *lingua franca* of many progressive economists today, it is the *lingua franca* of global health policy advocates. Speer, Fagan and Glozin, for example, write that a “post-pandemic recovery must be anchored in economic growth and productivity while including newer policy thinking around inclusivity”. They argue that ramped up economic growth is a “crucial precondition for addressing many of the challenges facing our society” from “funding for education, health, care and social services” to improving “employment, wages, and, ultimately, living conditions” [38]. This ‘Build Back Better’ approach promises stronger and more resilient health systems; a commitment it is incapable of delivering on. Although modern policy playbooks like the Sustainable Development Goals, a Pandemic Treaty and a European Green Deal might be participatory-oriented, well-being focused and environmentally conscious, they will, as pointed out by Labonté, inevitably bump up against the limits of our planetary ecosystem and a capitalist economy predicated on a continuous upward spiral of growth, (over) production, and (excess) consumption [39]. Even the WHO Council on the Economics of Health for all, which recommends that health policies move beyond GDP growth as a goal in itself, still argues that “health and wellbeing goals require investment and innovation by all actors in the economy, which can also help steer the rate and direction of economic growth” [40]. Growth-based green policy agendas sustain a global economic order in which wealthy nations continue to inequitably consume and exhaust most of the world’s natural resources, just as they did with COVID-19 vaccines [41]. They will do too little to redress structural global health injustices and will perpetuate colonial inequalities [9].

## Post-growth policy proposals for a caring economy

Most foundationally, a post-growth economy is one that abandons GDP growth as an objective and instead reorganises production around societal and planetary well-being rather than around consumption and accumulation. Through analyses and insights from ecological and feminist economics, post-growth policies focus on equitably meeting human needs within planetary boundaries and acknowledging the role of the informal economy in doing so by measuring and rewarding its contributions [42]. For instance, it is estimated that unpaid care work constitutes 16.4 billion hours a day, the equivalent of 2 billion people working 8-hour days without remuneration, three-quarters of which is performed by women [43]. Post-growth policies aim to reduce this imbalanced gender distribution of unpaid care work and harness the potential for care work to be turned into decent employment opportunities for all. According to Tim Jackson, “the care economy is the blueprint for a post-growth economy”, where we reconceive “economy as care” and prosperity as health rather than wealth. Central to this concept is “protecting the rights, wages and living conditions” of all care workers [44]. While providing a comprehensive set of post-growth policies goes beyond the scope of this article, key post-growth policies include the expansion and de-commodification of universal public services (including health, education, energy, transport, housing, food, and water); full employment through job guarantees; public works programs for the just transition to renewable energy; the democratic scaling down of less necessary and destructive forms of production; reduced working hours and improved workers’ rights; wealth taxes and other fiscal policy reforms; targeted industrial policy while expanding monetary sovereignty to ramp up public investments (e.g. the Bank of England, the UK’s central bank, created money to respond to the COVID-19 pandemic) [42, 45]. In relation to the 2023 conference at the European Parliament on how to move “beyond growth”, 400 civil society groups and experts have called for implementation of a set of beyond growth policies based on the four principles of Biocapacity, Fairness, Wellbeing for All and Active Democracy [46].

Critically, countries in the global South need to be able to mobilise production capacity around meeting human and planetary health objectives rather than serving consumption and accumulation in the global North. Key measures are required to address this extractivist arrangement and liberate labour and resources in service of local objectives. They include the elimination of sovereign debts that undermine health and well-being; ending structural adjustment conditions, which continue to enforce austerity and fiscal and monetary ‘discipline’ (i.e. conditions that prevent deficit spending); global tax cooperation to address tax evasion and resource theft from global South countries (e.g. the UN Convention on Tax as called for by the Africa Group); the democratisation of international financial and trade institutions (namely the International Monetary Fund, the World Bank, and the World Trade Organization); and ending unequal exchange (i.e. Northern appropriation of Southern labour and resources through trade) [26, 27, 47–49]. Practitioners, advocates and activists in global health must incorporate these analyses, ideas, and policy proposals as core to their work if they are serious about human and planetary health and wellbeing.

## Conclusion: Towards a true decolonial eco-just health agenda

As the ecological crisis further jeopardises fundamental determinants of health, planetary health thinking is gaining traction. Although rooted in principles like ecological sufficiency and global justice, a thorough historical and political economic analysis within the planetary health project is missing, and growthism often continues to prevail [8]. Meanwhile, talks about decolonising the global health field are becoming ubiquitous. But decolonisation is not a metaphor [20].

As Bluwstein has written, economic growth and a capitalist mode of production do not simply go away in a ‘transformed’ hypothetical future where knowledge and science are decolonized, and where marginalised perspectives on socio-ecological crises are recognised [50]. A decolonial eco-just global health agenda requires a material decolonisation of our global economic structures and arrangements, which is precisely what post-growth policies aim to bring forth.

Today, over 50 years after the launch of the Limits to Growth report of the Club of Rome and the call for a NIEO, the project of decolonisation remains incomplete and ecological time is not on our side. Growth-defending political and corporate interests continue to manifest themselves as deeply entrenched within the global health governance landscape. Indeed, no post-growth advocate would argue that challenging these power structures, addressing commercial determinants of health and advancing a post-growth agenda will come easily. The problem is not that we don’t have the imagination or policy proposals to secure a dignified life for all humans and non-humans with respect for the integrity of our biosphere. Global health advocates must pressure decision-makers to reverse the neo-colonial trajectory of deteriorating planetary health at the mercy of growth, and usher in post-growth policies for an economy centred on care.

## Data Availability

not applicable.

## Abbreviations

CO2: Carbon Dioxide
COVID-19: Coronavirus Disease 2019
GDP: Gross Domestic Product
NIEO: New International Economic Order
UK: United Kingdom
UN: United Nations
WHO: World Health Organization
